# Realistic monkey body animation reveals an uncanny valley in macaque body perception

**DOI:** 10.1371/journal.pbio.3003880

**Published:** 2026-07-14

**Authors:** Lucas M. Martini, Anna Bognár, Rufin Vogels, Martin A. Giese

**Affiliations:** 1 Hertie Institute for Clinical Brain Research (HIH) and Centre for Integrative Neuroscience (CIN), University Clinic Tübingen, Tübingen, Germany; 2 International Max Planck Research School for Intelligent Systems (IMPRS-IS), Tübingen, Germany; 3 Department of Neuroscience, KU Leuven, Leuven, Belgium; 4 Leuven Brain Institute, KU Leuven, Leuven, Belgium; Oxford University, UNITED KINGDOM OF GREAT BRITAIN AND NORTHERN IRELAND

## Abstract

Social interactions are essential for survival in primates, relying on both facial expressions and body signals. The accurate characterization of these signals is critical for understanding the neurocomputational mechanisms underlying social communication. While previous work has focused on recognizing monkey behavior, a causal and direct manipulation of individual cues strongly benefits from believable, dynamic body avatars—analogous to those successfully developed for faces. Creating lifelike monkey avatars with realistic body motion, however, is challenging. Acquiring sufficiently accurate movement data for animation with marker-based motion capture is impractical, and markerless tracking methods require extensive manual labeling. To address this, we developed MacAction, a realistic macaque body avatar animated from multi-camera markerless tracking data. Our method reconstructs accurate trajectories for a large number of keypoints, as required for the 3D animation of realistic body models. The entire time course of individual actions is captured using only two labeled keyframes per second, with performance further validated on a large-scale human multi-view dataset. We assessed the animation quality of our dynamic avatar in a free-viewing experiment with eight macaque observers for single macaque actions, where fixation behavior was indistinguishable between our animations and matched real videos. Moreover, by systematically varying the realism of the avatar, we found an uncanny valley effect in macaque body perception, similar to that previously described in both humans and macaque faces. These findings support the commonalities of social vision across primate species, providing a foundation for controlled experiments aimed at clarifying the detailed neurocomputational mechanisms of social body perception in primates.

## Abstract 2

Social interactions in primates rely on both facial expressions and body signals, but creating lifelike monkey avatars with realistic body motion is challenging. This study presents MacAction, a realistic macaque body avatar animated from multi-camera markerless tracking data, and reveals an uncanny valley effect in macaque body perception, similar to that described in humans.

## Introduction

Because of their genetic and physiological similarities to humans, rhesus macaques are the most widely studied nonhuman primate (NHP) species in biological disciplines, such as neuroscience, psychology, ethology, behavioral ecology, and medicine [1–4]. Scientific results from macaques have also been fundamental for understanding many biological functions in humans [5,6], and specifically human vision [7–9]. Macaques are a major model species for understanding human social perception, since they allow studies at the single-neuron level within a visual system that is highly similar to that of humans. Understanding these processes is critical not only from the viewpoint of neuroscience but also due to the growing number of technical applications that rely on the perception of digital human bodies, including games, film, virtual try-on [10,11], the metaverse [12], and clinical applications [13].

However, the computational and neural mechanisms involved in processing bodies in the primate brain remain poorly understood [14–16]. To clarify these mechanisms, experiments in psychology and neuroscience typically expose humans or monkeys to visual stimuli that manipulate underlying visual features in controlled ways [17–19]. The difficulty in studying body perception lies in the generation of dynamic body stimuli that can be exactly controlled. Avatars of monkeys, unlike real-world videos of behaving animals, offer the advantage of precise control over all relevant stimulus features and timing. However, creating these stimuli is challenging. Accurate motion data for their animation is not easily obtained, and such synthetic stimuli must guarantee a high level of realism to ensure the ecological validity of experimental results. Although monkey face avatars have been used successfully in the study of dynamic facial expression perception [20,21], to date, there have been no attempts to create lifelike monkey body avatars with realistic motion.

The digital reconstruction of a macaque’s moving body is a challenging problem. As with other animals, the amount of available tracking data from monkeys is limited [22], and creating datasets comparable in size to those for humans is impractical [22–26]. Moreover, standard motion capture technology is not applicable for recording full-body motion of macaques. Positioning markers reproducibly on their dense fur is difficult, and the animals typically remove or destroy body-attached markers during grooming. In addition, attached markers might alter their natural behavior, making deep-learning based markerless video tracking the only viable solution.

Various datasets that capture poses of monkeys in single-view images have been published [27–30]. However, tracking the dynamic body of NHPs in 3D space remains difficult, as monkeys exhibit a high-dimensional pose space comparable to that of humans. Recent multi-camera approaches have succeeded at the reconstruction of small sets of 3D keypoints for behavioral analysis, exploiting setups with a considerable number of cameras and extensive hand-labeling [31–33]. Such small marker sets, however, are insufficient for the animation of realistic avatar models with many degrees of freedom (DOF). At the same time, extending these methods for larger marker sets with sufficient tracking accuracy would result in impractically high amounts of required hand-labeling.

The use of computer-generated stimuli still raises the question of ecological validity, and even stimuli that appear realistic to humans might not do so to monkeys. In fact, it has been shown that as the realism of artificial agents or robots is continuously increased, observers might actually dislike artificial stimuli with high but not perfect levels of realism. This effect has been termed the "uncanny valley" (UV) according to an early paper in robotics that has formulated this hypothesis (Mori [34,35]). This phenomenon has nowadays been exploited to assess whether graphics-generated stimuli or robots appear realistic enough [36–40], and an UV has also been demonstrated in macaque observers for the recognition of static [41] and dynamic monkey faces [21]. Whether this effect also exists for monkey bodies is unknown.

**Fig 1:**
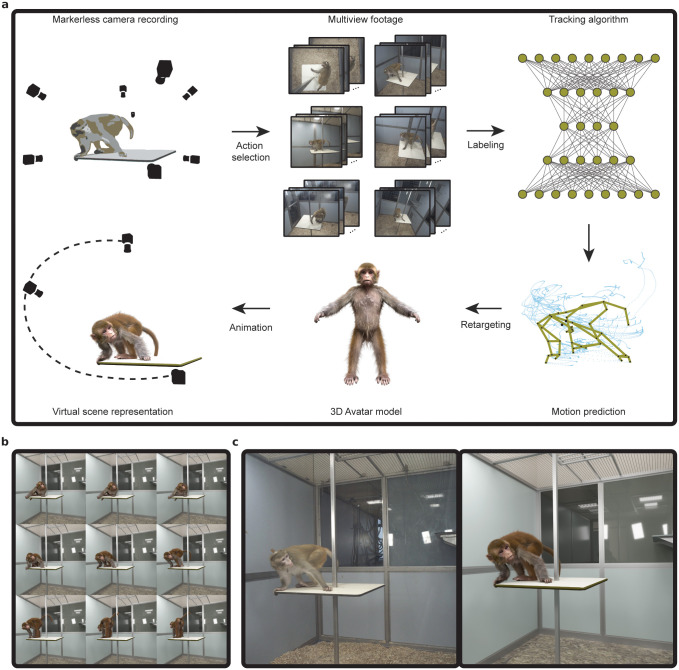
MacAction animates 3D markerless pose tracking to generate realistic synthetic images. **a,** Pipeline for character animation: selection of actions from sets of synchronously recorded videos from multiple viewpoints. A neural network-based tracking algorithm predicts keypoint trajectories for all frames of an action (shown in blue), exploiting a very small subset of hand-labeled keyframes for training. The retargeting process then maps this motion onto a 3D avatar. The resulting animation can be embedded into a 3D model of the background scene, which can be rendered from any viewpoint. **b,** Generated images of the virtual action over time. **c,** Comparison between corresponding frames from a real video (left) and the simulated virtual scene (right), combining the animated avatar with a synthetic 3D background scene.

Addressing the two key challenges of obtaining sufficiently accurate motion-tracking data from macaques for computer animation and generating animations with an appropriate level of realism, we have developed MacAction, a method for generating and validating highly realistic animations of macaque bodies based on markerless video motion capture. The core of our approach is a method for retrieving accurate 3D markerless pose trajectories for a large number of keypoints based on high-resolution multi-camera footage (Fig 1). It exploits learning-based methods in combination with different triangulation post-processing to reconstruct densely sampled action sequences from a small number of labeled keyframes. This enables the generation of realistic 3D animations of complex commercial avatars at high frame rates, requiring as few as only two labeled frames per second (LPS). Our method thereby substantially improves tracking accuracy compared to other macaque pose estimation methods in behavioral analysis [42], and we validate the realism of our animations in an eye fixation experiment with real monkeys. Not only do we show that the animals perceive our animations (S1 Video and S2 Video) as very similar to real-life video footage, but we also demonstrate the existence of an uncanny valley effect for bodies in NHPs. This makes our approach suitable for controlled investigations of the psychological and neurocomputational processes underlying body and social perception in primates.

In summary, our work makes the following contributions: a) MacAction combines markerless tracking and animation to produce realistic, dynamic body avatars that can also be reproduced by smaller laboratories with fewer cameras and limited manual labeling resources; b) we demonstrate, based on the fixation behavior of macaque observers, that our avatars are perceived as similar to videos of real animals—representing, to our knowledge, the first behavioral validation of dynamic body animations in NHPs; and c) our behavioral experiment provides the first evidence for an uncanny valley effect for bodies in NHPs, consistent with findings in human and in macaque face perception, suggesting shared principles of social body perception across primate species.

## Results

**Fig 2:**
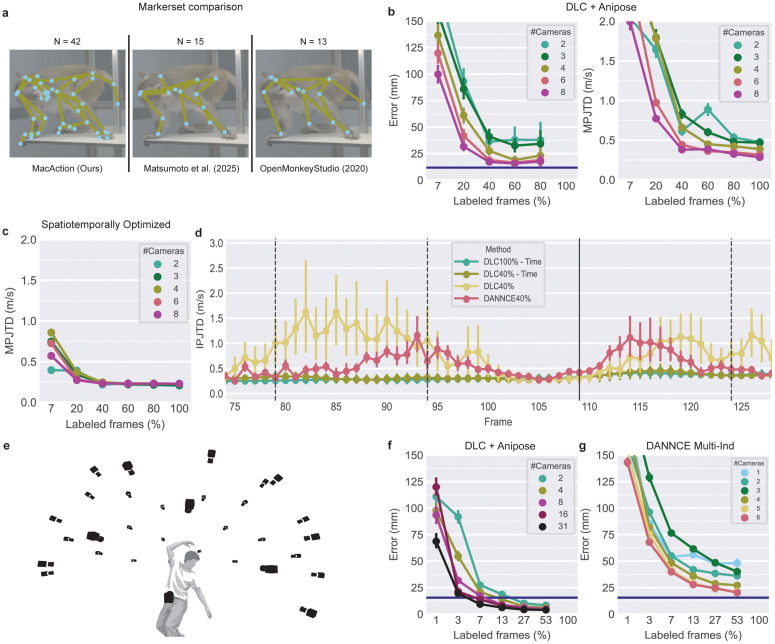
Single-action pose estimation. **a,** Comparison of the marker sets used in recent 3D behavioral tracking methods for macaques [31,32] with our approach. Markers are connected by lines representing the underlying skeleton model. **b,** Euclidean error (left) and smoothness measure mean per-joint temporal deviation (MPJTD) (right; Methods) for the entire sequence as a function of the number of labeled keyframes and cameras, using DLC + Anipose without spatiotemporal refinement for the submissive action. Errors are calculated from keyframes that were not part of the training data. The violet horizontal line indicates the spatial resolution (for macaques, 11.72 mm) of the volumetric approach, DANNCE. **c,** Smoothness measure MPJTD for all cameras and different fractions of keyframes used for training with optimally adjusted parameters for triangulation refinement. **d,** Time-resolved smoothness measure instantaneous per-joint temporal deviation (IPJTD) across the individual frames. Percentages denote the fraction of labeled frames used in training. For DLC-triangulated outputs, we included the optimized, smoother trajectories, indicated by “Time”, because spatial constraints were relaxed in the optimization results (panel a of Fig A in S1 Appendix). Vertical solid black lines indicate the labeled keyframes used for the training of all methods, while dashed lines indicate frames used for training only in the 100% conditions. **e,** Panoptic Studio camera setup [26]: a geodesic dome surrounds a human actor with 31 HD cameras. **f,** Euclidean error for tracking a dancing human actor with DLC using spatiotemporally optimized triangulation. DANNCE’s spatial resolution for human actions in f, g is indicated by the violet horizontal line (here, 15.625 mm). **g,** Euclidean error for the action interpolation with six human actors using DANNCE. DANNCE results are limited to six camera views because the pre-trained weights used for transfer learning were trained with six camera inputs. The data underlying this Figure can be found in https://doi.org/10.5281/zenodo.20490287.

To create realistic avatars that offer a high level of stimulus control, we devised a pipeline for the animation of a commercial macaque avatar model with 86 joints (a total of 261 DOF, including global translation) by exploiting markerless tracking. Realistic animation necessitates accurate 3D tracking of a sufficient number of keypoints in order to constrain this high-dimensional body pose (e.g., rotations of the arms, wrists, feet, finger movements, and the tail). In our work, we used 42 keypoints, a number that exceeds by factor two the one in previous studies on 3D markerless tracking of macaques [31,32] (Fig 2a), and 2D behavioral tracking in macaques [27,28]. As a comparison, typical marker sets in industry-standard marker-based motion capture for humans start with about a comparable amount of markers (Vicon Plug-in Gait [43]: *N* = 39, Qualisys Sports [44]: *N* = 43, Vicon Shogun [45]: *N* = 53) for body movement analysis (excluding fingers and facial movement).

State-of-the-art markerless tracking methods for animals [46–48] require human annotators to label exemplary postures for the training of underlying neural networks. For computer animation, however, the required amount of labeling would significantly escalate. Reducing manual labor is thus a key challenge in making our approach feasible for applications in neuroscience. To simplify the labeling process in multi-camera footage, we developed a software tool, AnimLabel, that propagates constraints from already annotated views to unlabeled ones.

The resulting pairs of multi-camera images and 3D poses served as training examples for state-of-the art markerless tracking algorithms, which are dedicated to reconstructing 3D poses of the intermediate unlabeled time points. We first tested DeepLabCut (DLC) [46,49], a popular image-based tracking algorithm for different animal species. To generate 3D poses, we processed tracked 2D keypoints from multiple views, exploiting the software Anipose [50]. This allowed us to incorporate spatiotemporally regularized triangulation that constrains 3D keypoints by optimizing their projections across views, their temporal smoothness, and minimizing the variance of their distance to neighboring markers. Specifically, two control parameters, $\alpha_{time}$ and $\alpha_{limb}$, determine the influence of smoothness of the joint angle trajectories and of length variations of the fitted limbs in the minimized cost function for this triangulation (cf. Materials and methods).

We also applied another approach, originally developed for human tracking [51], which integrates the triangulation process into the learning objective of the neural network. Its adoption for animals, the 3-Dimensional Aligned Neural Network for Computational Ethology (DANNCE), outperformed DLC with the same number of cameras for behavioral tracking in rats and mice [48]. The tracking results obtained with these two methods were further processed separately and mapped onto the skeleton of the macaque avatar. For this purpose, we used custom scripts in Maya for skeleton retargeting, making the pipeline adaptable to different marker configurations. Using smoothed 3D marker trajectories, we computed the avatar’s 3D poses for each time step and animated the actions in a virtual scene at high frame rates (Fig 1a, 2b). To ensure realism, we also reconstructed the static background scene in computer graphics based on laser scans of the real monkey cage (Fig 1c).

### Accurate single-action tracking with minimal labeling

Previous work on behavioral analysis in macaques has relied on considerable numbers of cameras and large amounts of labeled data to achieve markerless motion capture [31,32]. However, both camera systems and manual labeling of keypoints are costly resources, posing challenges for smaller laboratories and for applications in computer animation. Therefore, we assessed tracking quality as a function of camera count and labeling effort. Because the amount of training data scales with the number of cameras in triangulation-based methods (unlike volumetric approaches that combine multi-camera images into a voxel-based representation), we compared the aforementioned widely used behavioral tracking approaches: (a) DLC + Anipose, as an advanced triangulation approach; and (b) DANNCE, a volumetric method that learns directly from 3D skeletal poses instead of 2D keypoints.

#### For macaques.

For a complex submissive action, naive triangulation (also referred to as structural refinement; Materials and methods) with DLC + Anipose achieved a reasonable test error performance (mean error and SD, 18.9±20.8 mm; Fig 2b left) when trained on 40% of the action’s training data, corresponding to 1.8 LPS. The volumetric approach DANNCE, by contrast, did not perform as well even when trained with 80% of the data or 3.6 LPS using the same number of cameras (29.3±25.9 mm; panel c of Fig A in S1 Appendix). However, DANNCE predicted less jerky pose trajectories across the full action (Fig 2d; S3 Video). To obtain smoother pose estimates over time, we optimized control parameters in Anipose’s spatiotemporal triangulation (panel a of Fig A in S1 Appendix). With this optimization, models trained on only 40% of the data achieved temporal smoothness comparable to those trained on the full dataset (Fig 2d). We quantified this smoothness using the predicted marker speed across the sequence, termed instantaneous per-joint temporal deviation (IPJTD; Materials and methods). To summarize smoothness across models, we computed the mean per-joint temporal deviation (MPJTD) [52], defined as the average IPJTD over time for a given action.

For optimized triangulation, small amounts of labeled data were sufficient for models trained with different numbers of cameras to produce smooth actions (Fig 2c, 2d; S3 Video). At the same time, this approach maintained low tracking errors and yielded higher temporal consistency than DANNCE (Fig 2c and panels b and c of Fig A in S1 Appendix). Similar results were obtained for neutral walking behavior when trained with 5 labeled keyframes (corresponding to 71% of the available training frames, 2 LPS; panel d of Fig A in S1 Appendix). We also tested whether this approach is suitable for the simultaneous tracking of two monkeys. The attempt of multi-animal tracking with DLC failed, as the method could not reliably separate keypoints belonging to different animals when trained with limited data (14 labeled frames for 93.4 s; Materials and methods). Therefore, we resorted to DANNCE, which solves this assignment problem implicitly, as the 3D center of mass (COM) predictions of the individuals are provided to the algorithm. In practice, these COMs are also estimated by triangulation and training an auxiliary, separate keypoint predictor. With more labeled keyframes and additional cameras, DANNCE’s 3D keypoint errors decreased substantially (panel e of Fig A in S1 Appendix) but remained higher than for single-animal tracking, potentially reflecting the more complex body configurations and frequent self- and inter-animal occlusions present in the interaction behavior.

Taken together, these results demonstrate that optimized spatiotemporal triangulation enables accurate and temporally consistent markerless tracking of freely behaving macaques using only sparse manual annotations, while highlighting the additional challenges posed by multi-animal interactions.

#### For humans.

To verify that our method transfers from monkeys to another species and to leverage a dataset providing high-quality markerless motion from many color cameras, we also validated our tracking approach on human data. For this purpose, we extracted two dynamic actions (90 frames each, 3 s) performed by a single-actor (Fig 2e) and a group action involving six individuals from the large-scale human dataset Panoptic Studio [26], which provides simultaneous recordings from up to 31 cameras. In this dataset, each keyframe of an action was associated with ground-truth annotated keypoints in 2D and 3D, simulating up to 30 LPS.

For single-actor tracking, DANNCE trained with six cameras and half of the labeled frames achieved an average error of 37.9±33.5 mm (panel g of Fig A in S1 Appendix). In comparison, DLC with optimized spatiotemporal parameters resulted in prediction errors below the resolution limit of DANNCE, which is given by the discretization size of its input volume (15.625 mm; Fig 2f). This was true even for small numbers of cameras and labeled keyframes. For DANNCE, the mean marker speeds matched those of the ground-truth labels only when large amounts of training data were provided (panel g of Fig A in S1 Appendix right). In contrast, for DLC with spatiotemporally optimized Anipose triangulation, even small amounts of training data resulted in low MPJTDs comparable to the ground-truth level (panel f of Fig A in S1 Appendix). For tracking multiple individuals with our approach, DANNCE errors were lower than for tracking single individuals (20.3±20.2 mm; Fig 2g). This is likely explained by the lower action complexity and the fact that six times as many labels were annotated per keyframe in the multi-animal dataset. In this case, the ground-truth mean marker speed was matched using significantly less training data, likely because annotating six actors results in a much larger number of labeled 3D poses per keyframe (panel h of Fig A in S1 Appendix).

Consistent with our findings for macaques, tracking a single-action with limited data was more accurate using optimized triangulation of 2D keypoints with DLC + Anipose. Likewise, including more cameras and keyframes significantly reduced tracking error for multiple agents in the DANNCE version of our method. As a result, our approach shows that it is possible to achieve sufficiently accurate tracking for single-action sequences with as few as 2 LPS (corresponding to 6.66% labeled frames, or six labeled keyframes for a 3-s sequence).

These results demonstrate that our method generalizes across species and achieves robust 3D pose reconstruction on an independent large-scale benchmark dataset using only a small number of labeled keyframes.

### Animation of highly realistic monkey avatars using the MacAction pipeline

Building upon the generated accurate motion trajectory data, we were able to animate a highly detailed commercially available monkey avatar. This surface model comprised 261 DOF, 54,806 vertex points, high-resolution textures, and fur. For computer animation, we utilized the software Maya, retargeting the model’s underlying skeleton to pose trajectories with customized scripts and software bindings to Python (Materials and methods). The trajectories were not further corrected to suppress foot skating, e.g., by applying inverse kinematics approaches, in order to maximally preserve the original movement dynamics. The developed pipeline can be straightforwardly transferred to other avatar models with different skeleton definitions.

**Fig 3:**
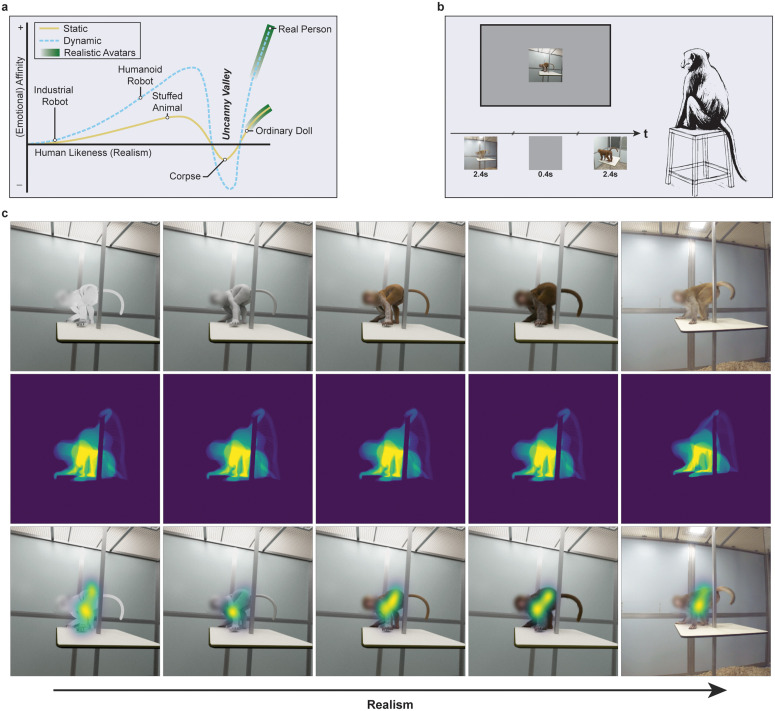
Uncanny valley experiment. **a,** Illustration of the uncanny valley hypothesis as originally formulated for human body perception, adapted from [35]. The range of highly realistic avatars or agents, both static and dynamic, is shaded in dark green. **b,** Experimental paradigm. **c,** First row: stimuli presented in the uncanny valley experiment with increasing degrees of realism from left to right. The rightmost stimulus is a naturalistic video. Second row: superposition of the monkey silhouettes (for degraded stimuli derived from the avatar with fur) across the same frames. Bottom row: accumulated fixations of the eight monkey subjects falling into these dynamic masks across all trials.

Making such artificial body stimuli useful for studying body perception in animals requires that monkeys perceive these stimuli as realistic. To verify this property, we conducted an ‘uncanny valley’ (UV) experiment (Fig 3a, 3b), where the perception of avatar animations with different levels of realism was compared with corresponding recorded movies of monkeys. The perception of the avatar as being highly realistic should result in a fixation behavior of the animals similar to that during the observation of real movies.

Therefore, we not only modeled a realistic monkey avatar and its dynamics, but also the background scene with 3D reconstructions of colored cage elements, real light sources, and reflections. Since body-selective neurons in monkey visual cortex often are tuned in a viewpoint-dependent manner [53,54], we rendered actions using seven different viewpoints that matched those of the real-world camera footage. These actions comprised the complex turning movement and the walk, representing submissive and neutral behavior, respectively. All generated dynamic stimuli were paired with a static version, in which a key pose most representative of the action was selected. The entire stimulus set comprised 140 stimuli, varying systematically the Action Type (submissive or neutral), the Render Type (level of realism; see below), the Viewpoint (7 levels), and the Presentation Type (dynamic versus static).

Consistent with many related animal studies in this field [21,41,55,56] we used eye fixation analysis to determine the attention and preference of animals for different visual stimuli. Such a relationship between eye movement and emotional preference is suggested also by numerous studies in humans (e.g., [38,57–60]). We recorded the eye movements of eight rhesus macaques while they observed avatar stimuli with different degrees of realism and matched real videos in random order, each for 2.4 s with an inter-stimulus interval of 400 ms (Fig 3b). In addition, we defined dynamic areas of interest (AOIs) based on the monkey silhouettes that were tracked in videos using the software Track-Anything [61], inspired by the definition of AOIs, such as eyes or nose, in face perception [21,55] (Fig 3c).

To test whether our avatar stimuli elicit visual responses comparable to real videos in monkeys, we first compared the fixation behavior for the nondegraded avatar stimuli with that for the matched real movies. Therefore, we analyzed the number of monkey silhouette fixations with generalized linear models (GLMs) assuming negative binomial distributions, following prior work [56]. We found significant influences for all experimental factors (Action, Render, Viewpoint, Presentation Type, Trial, and Individual; *p* < 0.0001; Table A in S1 Appendix) but no significant influence of the avatar (*p* = 0.96) or real videos (*p* = 0.51). The significant influence of trials (*p* < 0.0001) in the GLM model and a decrease in total fixation counts with an increasing number of trials (panel a of Fig B in S1 Appendix) may reflect a decay in the monkeys’ attention to the stimuli, as has been observed in UV face perception [55]. This was confirmed by fitting linear models to fixation counts for each individual, which showed negative slopes over increasing trial numbers in six out of eight subjects (Table B in S1 Appendix).

Accordingly, we also restricted this analysis to the first exposure of the monkeys to the stimuli. Again, the avatar and real stimuli did not significantly contribute to modeling (*p* = 0.60), nor did their interaction with the Presentation Type (*p* = 0.33) as evaluated by likelihood-ratio tests (LRTs) (Table C in S1 Appendix), i.e., the realism was preserved for dynamic presentations.

Summarizing, these results imply that our dynamic avatars elicited fixation behavior indistinguishable from that for real videos, suggesting very similar perceptual processing of the two stimulus classes.

### Uncanny valley in macaque body perception

Inspired by prior work on realistic human body perception [36–40], and work on face perception in monkeys [21,41,55,56], we asked the question of whether there exists an uncanny valley for body perception in monkeys. This would imply a U-shaped relationship between avatar realism and the animal’s preference for the stimuli. Following previous studies in face perception [21,41,56], we used the number of fixations and fixation durations as proxies to determine the monkeys’ preferences for different stimuli. Similarly, we modified the appearance of the avatar according to a previous face perception study [21], where the assumed order of perceived realism was confirmed by psychophysical ratings of human observers. Starting from the unmodified highly realistic avatar, we first removed the fur, then color and texture, and for the most unrealistic version, we presented a white wireframe texture, removing shadows and depth cues of the surface structure (Fig 3c). All versions of the avatar were animated with the same body motion, matching the motion in the corresponding real video.

**Fig 4:**
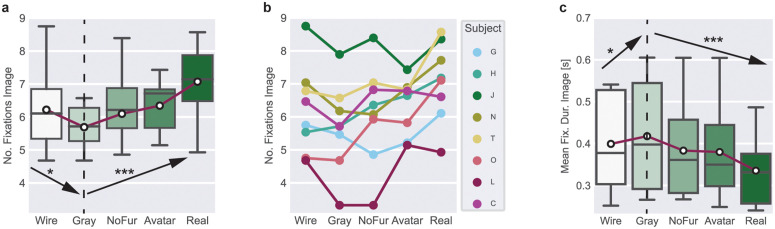
Uncanny valley in the body perception of macaques. **a,** Box plot of the number of image fixations for different Render Types. The plot in c shows results including all trials, while the panels a, b include the first trials only. **b,** Number of image fixations shown for the eight monkey subjects individually. **c,** Box plot of the mean image fixation duration, for each Render Type and including all trials. Box plots show the median with interquartile range (IQR), and whiskers at 1.5× the IQR. The arithmetic means are indicated by white circles. In a and c, arithmetic means are connected by a red lines. Statistically significant slopes of the Two-lines test are indicated by arrows and asterisks. The test was performed on centered data without aggregation: $p^{*}<0.05$, $p^{**}<0.01$, and $p^{***}<0.001$. The vertex points of the valleys in a, c is indicated by the dashed vertical line. The data underlying this Figure can be found in https://doi.org/10.5281/zenodo.20490287.

In order to analyze the existence of an uncanny valley, we investigated the fixation behavior again as a function of the Render Type (in this case including 5 levels of realism, from the wireframe up to the nondegraded avatar, and the corresponding real movie). First analyzing the number of fixations for first trials (Fig 4a) and collapsing across the other experimental factors, we observed a U-shaped relationship between the realism of the presented avatar (Render Type) and the fixation number, where the gray avatar was fixated least frequently. This is true for the average data, as well as for the data of several of the individual animals (Fig 4b).

An analysis of several low-level image features and general saliency rules out that the observed valley is explained by simple dependencies of low-level features or saliency on Render Type (see Fig C and Table G in S1 Appendix, and Materials and methods for details). This observation was further statistically confirmed across all animals in two ways.

Using our GLM analysis with likelihood-ratio tests, the Render Type had a significant influence on image fixations (*p* < 0.0001, Table D in S1 Appendix). While the U-trend is apparent in Fig 4a, one might argue that a significant influence of the factor Render Type in the GLM analysis just shows the existence of differences between the factor levels, but not necessarily a U-shape. For this reason, we additionally applied a specific nonparametric test for the existence of a significant U-shaped relationship [62]. This Two-lines test confirms the simultaneous existence of a negative trend for the left part of the functional dependence, and a positive trend for the right part, while simultaneously estimating the turning point of a valley or peak. Applying this test to the image fixation counts, collapsing the data across all factors except for the Render Type, we found a significant U-shape (Fig 4a, *p* < 0.05, *p* < 0.001, turning point: “Gray”). Consistent results for the fixation statistics were obtained in monkeys for faces [21,41]. In addition, studies in humans have also reported uncanny valley effects for eye movements measures, partly using decision paradigms [38,58].

When analyzing the fixation duration, a reversed uncanny valley was apparent, where animals looked longer at stimuli with an intermediate realism level (Friedman: $\chi^{2}(4,n=8)=11.8,p=0.019$, panel c of Fig B in S1 Appendix). Although the Two-lines test did not show a peak when only the first trial was considered, including all trials showed not only a significant effect but also significant slopes and the turning point at the gray avatar (Friedman: $\chi^{2}(4,n=8)=19.3,p=0.0007$; Fig 4c, *p* < 0.05, *p* < 0.001, turning point: “Gray”). The same effect was apparent for image fixations of all trials (panel b of Fig B in S1 Appendix, *p* < 0.05, *p* < 0.001, turning point: “Gray”). An overview of the Two-lines test results is given in Table E in S1 Appendix.

This inverse uncanny valley effect for mean fixation duration is consistent with the findings of [21], whereas [41] reported a conventional uncanny valley effect for cumulative fixation duration. These findings may initially appear contradictory, but under a fixed viewing period the cumulative fixation duration tends to be positively correlated with fixation count, whereas the mean fixation duration is expected to vary inversely with fixation count, such that fewer fixations tend to be associated with longer individual fixations. In fact, [21] found stronger effects for mean fixation duration than for cumulative fixation duration, which is consistent with our findings and motivated our focus on mean fixation duration as a measure of this effect.

Since the uncanny valley is expected to be stronger for dynamic stimuli, we further confirmed a significant influence of the Presentation Type (static versus dynamic; *p* < 0.0001, Table D in S1 Appendix) but did not find an interaction with the Render Type (*p* = 0.55). Similar results with a more subtle valley were found for monkey silhouettes (panel d of Fig B in S1 Appendix). Again, the influence of the Render and Presentation Type were significant (*p* < 0.0001 and *p* < 0.0001), unlike their interaction (*p* = 0.81, Table F in S1 Appendix). This implies that the observed uncanny valley is also present for static stimuli. Since the body motion was the same for all render types, except for the real video, the observed valley is not a result of changes in the body motion.

Summarizing, this analysis provides the first evidence for the existence of an uncanny valley for body perception in monkeys. The obtained results are similar to the ones of a previous uncanny valley study for faces that was based on a very similar paradigm [21]. Consistent with this study, the exact position of the valley varies slightly between different monkey individuals.

## Discussion

The investigation of detailed neural and computational mechanisms of social perception in primates ideally requires manipulable dynamic body stimuli that allow precise control over all relevant stimulus parameters while remaining realistic and ecologically valid. Natural stimuli fulfill the last requirements, but typically introduce a vast variety of uncontrolled features, many of which also influence visual processing, making it difficult to isolate specific computational functions with such stimuli.

As solution for this problem, we present MacAction, a method for the generation of lifelike animations of monkeys with realistic motion. Our method combines accurate 3D markerless motion capture, obtained from multi-camera footage while requiring only minimal hand-labeling, with the animation of a highly realistic macaque avatar model. Unlike in humans, no large databases with accurate 3D motion or video data are available for NHPs, which makes the standard approaches for accurate video-based tracking and marker-based body animation impractical in this context. Specifically, existing work on multi-camera tracking of animals and macaques has focused primarily on 3D behavioral analysis [31,32,50,63]. In contrast to these applications, our approach generates accurate tracking data with a sufficient number of keypoints that are necessary for the estimation of 3D joint angles of complex computer graphics models. The key to achieving this is that our method substantially reduces the required amount of manual labeling while maintaining accuracy. Compared to macaque behavioral tracking, our approach tracks single-actions more precisely, even relative to methods using richer sensor information such as RGB-D cameras [42,64], which report mean errors exceeding 3.7 cm. For tracking individual animals with limited data, we compared 2D multi-view tracking with advanced triangulation techniques and 3D volumetric pose estimation. While the 3D tracking approach was more reliable in multi-individual tracking, single-actions could be tracked more accurately with less data by the triangulation of 2D keypoint trajectories, effectively reducing the labeling amount for computer animation to only 2 LPS. Considering the known challenges of tracking macaques [28,31], our method is naturally adaptable to other animals with simpler kinematic structures. This is particularly interesting for studying dynamic actions of endangered animal species [65], where data may be scarce and costly to obtain. While we have tested the suitability of our method for tracking multiple animals within a scene, the performance and stability of tracking in multi-animal scenarios can still be substantially improved. Addressing this challenge will be an important problem for future research, especially for the efficient generation of data from dyads or groups of interacting animals.

MacAction goes significantly beyond motion tracking, as it also includes a pipeline for the creation of highly realistic dynamic avatars and their validation. Our work provides evidence through an experiment with real macaques that the animals’ fixation behavior toward our avatar closely matched that of real movies, suggesting a similar processing in terms of social perception. Obviously, the set of chosen behaviors in this paper was limited. However, the proposed methodology lays the groundwork for establishing a much larger stimulus set focusing on different classes of socially relevant behaviors. A substantially extended dataset that was established using the described methods was recently delivered by [66].

Uncanny valley experiments have been common to quantify the realism of human avatars in computer graphics [39,40], and the same behavioral measures have been used frequently for the quantification of the perceptual preference in monkey face perception [21,41]. An important biological result of our study is that we were able, for the first time, to demonstrate an uncanny valley effect for body perception in monkeys. In human body and face perception, such effects are well established [36–38], and an uncanny valley has also been reported in monkey face perception [21,41]. Our study provides important behavioral evidence that supports the similarity of the perceptual processes of body perception in humans and NHPs. This fact is critical for justifying NHPs as a valid model for human social perception [14,16].

## Materials and methods

### Animals and husbandry

Eight male rhesus monkeys (Macaca mulatta), ranging in age from 5 to 7 years, contributed to this study. The animals are housed in enclosures at the KU Leuven Medical School and experience a natural day-night cycle. Each monkey shares its enclosure with at least one other cage companion. On weekdays, dry food is provided ad libitum, and the monkeys obtain water, or other fluids, during experiments until they are satiated. During weekends, the animals receive water along with a mixture of fruits and vegetables. The animals have continuous access to toys and other forms of enrichment. For experiments unrelated to this paper, the monkeys were implanted with a plastic headpost, attached using ceramic screws and dental cement following standard aseptic procedures and under full anesthesia. Both the animal care and experimental procedures adhere to regional (Flanders) and European guidelines and have been approved by the Animal Ethical Committee of KU Leuven (protocol number 182/2019).

### Multi-camera recording setup

We created a 3D model of the surrounding scene in the animal house using a commercial imaging laser scanner (LEICA BLK360). The resulting extracted colored point clouds from multiple scans were registered using Scasa PinPoint (v2.6.0), and further processed with Autodesk Recap 2022. The size of the surrounding cage was (2.1 m x 3.1 m x 2.3 m). Based on these scans, we reconstructed the entire surrounding environment as a 3D model in Maya 2022. A detailed protocol for the 3D laser-scanning and virtual scene reconstruction workflow, together with the final reconstructed Maya scene, is provided in the archived materials described in the Code and Data Availability section. The reconstructed scene elements were then imported into the real-time gaming platform Unreal Engine (4.27.1). In this software environment, we created virtual cameras to emulate those we planned to use in the final recordings. Adjustments to these cameras, including their extrinsic position and field of view (FOV), helped to choose appropriate lenses. We determined the focal length in relation to the film size in order to achieve a suitable FOV. We then verified that for the chosen lenses a virtual cube with a side length of 75 cm, which represented the macaque’s movement space, was fully captured in the FOV. This cube was centrally placed in the virtual cage, and it was captured at a resolution of 2056 × 2056 pixels by the virtual cameras.

For the real recordings, we employed eight high-resolution machine vision color cameras from IO Industries. Four cameras of the type Victorem 51B163CCX (2464 × 2056 pixels) and four of the type Victorem 120B68CCX (4112 × 3008 pixels) were initially positioned at the virtual camera locations. For synchronization and recording, we used two digital video recorders (CORE2CXPLUS) from the same manufacturer, enabling precise synchronization with low-latency TTL signals. Together with the cameras also eight LED light panels (StroboMini2, Norka Automation) were controlled by TTL signals. This allowed to lit the scene in synchrony with global camera shutters, reducing the overall brightness experienced by the monkeys significantly. As a result, the cameras’ exposure time could be set to as low as 300 μs, keeping motion blur effects at a minimal level. Four light panels surrounded the cage horizontally, and the remaining ones were placed on top of the cage next to a single camera, recording a top view. The cage’s top consisted of a metal grid on which a plexiglass plate was mounted, protecting the cameras from manipulations by the animals. The remaining equipment was mounted on tripods outside of the cage. Since the setup was not completely protected against mechanical perturbations, we re-calibrated the optical recording system regularly before each recording session using two different calibration schemes. The first scheme involved a custom ChArUco pattern (39.7 cm x 39.7 cm, 0.5 marker-to-square ratio, OpenCV standard 4-bit dictionary with 50 markers for ArUco checkers). This pattern was detected by software written in Python (3.9.7) using OpenCV (opencv-python 4.5.5.62 and opencv-contrib-python 4.5.5.62). Additionally, the extrinsics were optimized with bundle adjustment in MATLAB (R2021b). The second calibration method involved a light-emitting wand from Vicon (Active Wand), developed for the calibration of a commercial marker-based motion capture system, and part of proprietary post-processing software. Both methods achieved subpixel accuracy. After the recordings, we transferred raw video material at high speeds to an external storage system (D5 Thunderbolt 3, Terramaster) with up to 90 TB storage capacity.

### MacAction software

MacAction was developed using Python 3.9.7 and incorporates several widely used open-source software packages for the scientific community: numpy 1.22.1, aniposelib 0.4.3, matplotlib 3.5.0, pyqtgraph 0.11.0, opencv-contrib-python 4.5.5.62, opencv-python 4.5.5.62, and scipy 1.7.3. We employed established keypoint estimation software, anipose 1.0.1, deeplabcut 2.2.2 (cuda 11.4 and cudnn 8.2.1) and dannce 1.2.0 (cuda 10.1 and cudnn 7.6.5). Our repository includes code modules for various functions: video compression, gamma correction, camera calibration, and calibration file conversion. We further developed and provide the package AnimLabel, a labeling tool to generate 3D poses from simultaneously presented multi-view images. AnimLabel enforces consistent labeling across an arbitrary number of views by including multi-view geometric constraints from calibration data. Epipolar lines are utilized to reduce relevant regions across views to a single line or intersections of lines if a keypoint is labeled in a single or multiple views. To further reduce the labeling effort, we project triangulated landmarks of at least two views into all other perspectives.

In practice, we use at least three hand-labeled markers to reduce the uncertainty of the triangulated 3D keypoint positions.

In this way, labeling effort remains the same, but labeling throughput scales linearly with the number of cameras. We also ensure sufficient labeling accuracy by setting a threshold for the mean reprojection error across labeled views (5 pixels).

The software provides the possibility to crop views, showing only relevant image areas, which is especially relevant for high-resolution images. AnimLabel can also export cropped single-animal actions per view. Based on a pre-defined spatial margin, we project all markers into the image plane. We then identify the markers that are most distant from the center of the frame to construct a cropped frame that does not clip any of the body regions.

Data labeled through AnimLabel, can be exported in appropriate formats to both, DANNCE and DeepLabCut for multi-view and multi-animal projects. We convert the tracking data from DeepLabCut to Anipose’s format for single- and multi-animal tracking.

All generated full-body trajectories are then saved in a numpy binary format (’.npy’). For rendering, we use Maya, leveraging its native Python 3 interpreter, mayapy, which allows to import and retarget motion data. Maya allows to enhance the quality of the motion capture data by interpolation to smoother trajectories, and we exploited calibration data to align virtual cameras with their real-world counterparts.

### Datasets

Our study generated or used the following datasets:

*Macaque animation.* Video data of macaques was recorded at 80 frames per second (FPS). From 2.5 hours of recording data, we extracted three distinct actions: a submissive action (3.43 s), a neutral action (2.44 s), and an interaction of two macaques (93.4 s) with 15, 7 and 14 labeled frames, respectively. Because the longer grooming interaction required annotation of two animals and contained repetitive motion, it was labeled less densely in time. Based on these labeled frames, we assessed the accuracy of our tracking pipeline in three different scenarios: (1) oversampling a short, complex body movement; (2) providing fewer labels for a concise, simpler action; and (3) fewer labeled time points of a prolonged multi-animal close-interaction sequence.

We labeled an extensive set of 42 body landmarks: ‘Jaw’, ‘lowerLip’, ‘upperLip’, ‘lCornerM’, ‘rCornerM’, ‘Nose’, ‘lEye’, ‘rEye’, ‘Head’, ‘lEar’, ‘rEar’, ‘Neck’, ‘lShoulder’, ‘lElbowOut’, ‘lElbowIn’, ‘lWristOut’, ‘lWristIn’, ‘lThumb’, ‘lMidK’, ‘lMidTip’, ‘rShoulder’, ‘rElbowOut’, ‘rElbowIn’, ‘rWristOut’, ‘rWristIn’, ‘rThumb’, ‘rMidK’, ‘rMidTip’, ‘midSpine’, ‘lHip’, ‘lKnee’, ‘lAnkle’, ‘lBigToe’, ‘lFootTip’, ‘rHip’, ‘rKnee’, ‘rAnkle’, ‘rBigToe’, ‘rFootTip’, ‘beginTail’, ‘midTail’, and ‘tipTail’. This allowed us to infer both the positions and rotations of individual joints in 3D, crucial for controlling a high-quality virtual avatar.

Due to the significant amount of work involved in labeling markers on larger data sets, we limited the number of keypoints to the minimum necessary for animating the chosen avatar model.

*Human benchmark*. To evaluate the amount of labeling required for multi-view and cross-species tracking, we also utilized a publicly available large-scale data set for humans, the CMU Panoptic dataset [26]. This data set provides ground-truth data obtained with a large number of cameras, and it has significantly contributed to the development of human pose estimation algorithms, and specifically in volumetric pose estimation [51], forming the basis of the DANNCE method [48].

We refrained from evaluating our method on datasets constructed with physically attached markers ([67,68]). These keypoints can become salient in images and skew the learning process for markerless tracking. In contrast, the Panoptic Studio dataset provides motion capture data without physical markers, utilizing recordings from 480 VGA, 31 HD, and 10 Kinect v2 RGB + D cameras, evenly distributed in a geodesic dome. Designed to capture human social interactions, it comprises actors engaged in activities such as playing games and instruments, or showing various other types of movements. For comparison with our monkey dataset, we extracted short sequences from movies showing two actions: a complex dance movement, and a clip involving six individuals engaged in simultaneous interactions. Each action consists of 90 frames lasting ~3 s. We used data from up to 31 HD cameras at a resolution of 1920 × 1080 pixels.

The volumetric approach of the DANNCE algorithm has the disadvantage of a limited tracking volume. In order to better align the algorithm’s accuracy between humans and macaques, we restricted the included 3D poses to upper body keypoints, and hips. Consequently, the human marker set comprised the following 15 keypoints: ‘Neck’, ‘Nose’, ‘BodyCenter’, ‘lShoulder’, ‘lElbow’, ‘lWrist’, ‘lHip’, ‘rShoulder’, ‘rElbow’, ‘rWrist’, ‘rHip’, ‘lEye’, ‘lEar’, ‘rEye’, and ‘rEar’. Finally, we sampled actions over time and across different numbers of cameras to create diverse datasets for benchmarking the tracking algorithms.

### Pose estimation

The pose estimation pipelines incorporated in this study specifically address single-animal pose estimation. Therefore, we fine-tuned a pre-trained ResNet-101 model on the ImageNet dataset using default training configurations in DeepLabCut 2.2.2. We created the dataset by exporting actions from AnimLabel, downsampling the high-resolution images by a factor of two for computational efficiency.

After training and inference on a particular view, we consider all 2D keypoint predictions, regardless of their confidence values. In this way, we do not restrict information prior to the triangulation process. We use Anipose’s Random Sample Consensus (RANSAC) triangulation method and optimize the 3D points by spatiotemporally regularized triangulation, an extension that incorporates not only the reprojection error [50]:


$$\begin{array}{c} ℒ=L_{\text{proj}}+\alpha_{\text{time}}L_{\text{time}}+\alpha_{\text{limb}}L_{\text{limb}}, \end{array}$$

where the respective loss *L*_proj_ aims at minimizing the distance between the projected 3D markers and the associated 2D predictions over cameras. Since 3D body marker trajectories should be smooth in time and ensure consistent limb lengths, the losses *L*_time_ and *L*_limb_ are added, which measure the mean joint deviations over time and with respect to the expected limb lengths between connected keypoints. The parameters $\alpha_{i}$ in the composite loss allow to adjust the importance of these different factors in the cost function. We refer to the triangulation algorithm for the choice $\alpha_{\text{time}}=\alpha_{\text{limb}}=0$ as structural refinement, as initial 3D points are optimized and shifted only to yield a consistent projection across views.

For DANNCE, we fine-tuned pre-trained weights of their AVG network architecture on a sixcamera setup for rat pose estimation. Note that these pre-trained weights effectively limit the number of camera views that can be evaluated to six. As with DeepLabCut, we created custom scripts to align the labeling structure with AnimLabel and used the default training configurations. The algorithm requires a prediction of the animal’s 3D COM, which was determined using quadratic B-spline interpolated hand-labeling (of the ‘midSpine’ for macaques, and ‘Neck’ for the upper human body), instead of training a separate COM network within DANNCE. We specified the encapsulating volume size by determining the volume that encompassed all individuals and poses in the dataset, adding an additional safety margin. We did not incorporate any temporal or spatial smoothing, except for potential interpolation of keypoints in the commercial animation software.

*Error metrics.* To quantify the position accuracy of the tracking, we use the Euclidean distances between the predicted and the ground-truth 3D joint positions. As in [51,52], we computed the mean per-joint position error (MPJPE):


$$\begin{array}{c} \text{MPJPE}(\text{J},\hat{\text{J}})=\frac{1}{N_{J}}\sum_{j=1}^{N_{J}}\|{\text{J}}_{j}-{\hat{\text{J}}}_{j}{\|}_{2}, \end{array}$$

where the vector **J** includes the $N_{J}$ ground-truth 3D keypoint coordinates, and $\hat{\text{J}}$ denotes the corresponding tracking estimate. We average this per-frame error across the entire action to provide an overall error measure for the sequence. In addition, we compute Euclidean errors for specific body parts in the same way, including only the landmarks of those body parts.

Another measure characterizes the smoothness of the tracked trajectories. If the tracking fails, typically the trajectories show jumps and nonsmooth transitions, for example, when the triangulation procedure misaligns 2D keypoints across cameras. Inspired by [52,69], we define a measure for the temporal smoothness of the predicted action of length *T* by the MPJTD:


$$\begin{array}{c} \text{MPJTD}(\hat{\text{J}})=\frac{1}{T-1}\sum_{t=1}^{T-1}\text{IPJTD}(\hat{\text{J}}), \end{array}$$

where the IPJTD is the action’s average of the IPJTD:


$$\begin{array}{c} \text{IPJTD}(\hat{\text{J}})=\frac{1}{N_{J}}\sum_{j=1}^{N_{J}}\|{\hat{\text{J}}}_{t,j}-{\hat{\text{J}}}_{t+1,j}{\|}_{2}. \end{array}$$

The IPJTD can be thought of as the instantaneous speed, or first-order derivative, of the predicted keypoints. We found that large values of this quantity are typically caused by jumps in the tracking, i.e., by artifacts, and not by veridical fast body movements.

### Animation

*Model.* We utilized a commercial macaque avatar model from CGTrader (https://www.cgtrader.com/3d-models/animal/mammal/realistic-monkey-fur-rigged). This digital macaque model consists of a mesh with 54,806 vertices, includes 8192 × 8192 pixel resolution textures, and fur. It is fully rigged, i.e., linked to a hierarchical structure of 86 joints that allows for posing the character with appropriate deformations of the surface structure. It uses linear blend skinning to determine these surface deformations. To enhance the realism of the avatar, we scaled hands and feet to match measurements of one real monkey, derived from triangulated poses of hand-labels. Preliminary eye tracking results indicated that the animals were specifically interested in the rear part of the observed bodies. Consequently, we refined this area of the model by attaching a pair of testes and two patches of keratinized skin (sciatic protuberances), characteristic of Old World monkeys, using rivet constraints in Maya 2023. Additionally, we utilized the XGen Interactive Groom Editor to modify the fur in this area, aiming for a less dense and shorter fur texture.

*Pipeline.* Utilizing the tracked 3D keypoint trajectories of all markers, we implemented a customized yet generally applicable interface to the kinematic tree of a digital avatar model in Maya. The keypoints are rescaled to match the dimensions of the avatar, and the character’s joint positions and rotations are set to their default states, known as the rest pose. We then position the root joint of the avatar in the scene, and determine the rotations of individual joints along its kinematic tree by solving Euler angle transformations in matrix form consecutively. A detailed description of the approach is given below. For body segments in the avatar with more intermediate joints than tracked keypoints, that is, the spine and tail, we perform a quadratic B-spline interpolation, treating these joints as having two DOFs.

*Posing single joints in the avatar model.* We distinguish between joints with two and three rotational DOF. For joints of two DOF, consider the column vector $a=(a_{1},a_{2},a_{3}{)}^{T}$ as the relative position of the succeeding element in the avatar’s kinematic tree. We denote $b=(b_{1},b_{2},b_{3}{)}^{T}$ as the respective tracked version of *a* in the same coordinate system. After transforming both vectors into spherical coordinates $\left(r_{x},\theta_{x},\varphi_{x}\right)$, we subtract their angles defining the angle differences $\varphi_{diff}=\varphi_{b}-\varphi_{a}$ and $\theta_{diff}=\theta_{b}-\theta_{a}$. We construct a rotation matrix around the z-axis and rotate *a* by it with $\varphi_{diff}$. With this transformed vector and b, we construct the orthogonal vector $b\times aR_{z}(\varphi_{diff})$ by forming the cross-product. We normalize the resulting vector and denote it as *e*. To rotate *e* by the angle $\theta_{diff}$, we use the matrix form of an axis-angle rotation. In general, the counter-clockwise rotation around an unit vector $u=(u_{1},u_{2},u_{3}{)}^{T}$ by an angle α is given by [70]:


$$\begin{array}{c} R_{u}(\theta )=[\begin{array}{ccc} q+u_{1}^{2}\left(1-q\right)u_{1} & u_{2}\left(1-q\right)-u_{3}pu_{1} & u_{3}\left(1-q\right)+u_{2}p\\ u_{2}u_{1}\left(1-q\right)+u_{3} & pq+u_{2}^{2}\left(1-q\right)u_{2} & u_{3}\left(1-q\right)-u_{1}p\\ u_{3}u_{1}\left(1-q\right)-u_{2} & pu_{3}u_{2}\left(1-q\right)+u_{1} & pq+u_{3}^{2}\left(1-q\right) \end{array}]\\ \text{with}\;p=\sin\theta \;\text{and}\;q=\cos\theta . \end{array}$$

Therefore, the transformation of *a* to *b* can be written as $R_{1}=R_{e}(\theta_{diff})R_{z}(\varphi_{diff})$. We adjust the sign of $\theta_{diff}$ according to the orientation of *e* to rotate in the appropriate direction. This technique establishes the pose for joints with two DOFs, keeping the roll of the transformed *a* unchanged. The two transformations relate to a yaw and pitch transformation of the animated vector.

For three-DOF joints, each vector—both animated and tracked—relates to a secondary vector required to define the roll. Here, *c* and *d* denote the secondary vectors for animation and tracking, respectively. Note that the rotations between *a*, *c* and *b*, *d* are generally not the same. Thus, we transform *c* by *R*_1_ and determine the roll that makes $\begin{array}{c} R_{b}(\gamma )\;R_{1}c \end{array}$ coplanar with *b* and *d*:


$$\begin{array}{c} A=\left[\;b\;|\;d\;|\;R_{b}(\gamma )\;R_{1}\;c\;\right],\text{and}\\ \det A\overset{!}{=}0, \end{array}$$

where *A* denotes a column matrix composed of these three column vectors. The angles that satisfy this condition are given by:


$$\begin{array}{c} \gamma =\pi n-\arctan(\frac{x}{y})\\ \text{with}\;y\neq 0\;\text{and}\;n\in \mathbb{Z}, \end{array}$$

where *x* and *y* can be expressed as:


$$\begin{array}{c} x=(d_{2}c_{3}-d_{3}c_{2})b_{1}+(d_{3}c_{1}-d_{1}c_{3})b_{2}+(d_{1}c_{2}-d_{2}c_{1})b_{3},\\ y=(-d_{2}c_{1}-d_{1}c_{2})b_{1}b_{2}+(-d_{1}c_{3}-d_{3}c_{1})b_{1}b_{3}+(-d_{3}c_{2}-d_{2}c_{3})b_{2}b_{3}\\ +(d_{2}c_{2}+d_{3}c_{3})b_{1}^{2}+(d_{1}c_{1}+d_{3}c_{3})b_{2}^{2}+(d_{1}c_{1}+d_{2}c_{2})b_{3}^{2}. \end{array}$$

We determine whether we need to add π to the rotation by computing the scalar product with *d* and the transformed *c*. All other solutions represent the same two rotations.

### Benchmarking details

All benchmarking and training procedures were conducted on Nvidia Geforce RTX 3,090 and 4,090 GPUs. In the datasets, we prioritized cameras that increase view variability.

*DLC.* We trained a single model across views for each species. Single-animal models were trained for 5,000 epochs and multi-animal models for 1,000 epochs. All models utilized a ResNet-101 backbone. Data sets with more than five images were divided into 95% training, and 5% testing sets. After training, we predicted 2D landmarks on complete video sequences. These predictions were then converted into 3D trajectories using Anipose.

*DANNCE.* Our training followed the guidelines provided on https://github.com/spoonsso/dannce/wiki. We trained models across different animal types and dataset sizes, each for 1,000 epochs. Due to limited data sizes, we excluded validation samples. For all species, models were trained using a 64 × 64 × 64 volume grid, resulting in voxel resolutions of 11.719 mm (macaques) and 15.625 mm (humans), based on the cubic volume side lengths of 750 mm and 1,000 mm, respectively. For training configurations with less than six cameras, models trained on duplicated camera views.

### Uncanny Valley stimuli

For the behavioral experiment testing the quality of our animated macaque avatar, we adopted a similar approach as in experiments that showed the presence of an uncanny valley in macaques for faces [21]. According to the uncanny valley hypothesis, variations in the avatar’s realism should induce changes in preference, which can be discovered by eye tracking. Starting from the complete avatar model, we created three degraded versions of it: (1) discarding the fur of the model and depicting the avatar with skin texture only; (2) removing the color information of the skin by the replacement of a smooth gray texture; and (3) discarding light reflection properties of the surface structure by outlining the mesh’s edge lines in black on a constant white background. As actions for these stimuli, we used the emotionally neutral walking and the submissive body movement.

For the final animations, we also reconstructed the background scenery, including the cages and lights, as 3D scan-based models of the individual geometric elements in Maya. To reproduce the recording conditions virtually, we positioned rendering cameras according to their real-world counterparts. Due to the remaining shape difference between the real macaque and the avatar, the same motion can result in slightly different trajectories of the corresponding body parts, most apparent in the distal parts of the avatar’s limbs. To reduce these differences, we adjusted strongly deviating motion paths in Maya 2023.1 using the Graph Editor tool, ensuring a realistic physical interaction of the avatar with its environment.

Excluding the unnatural viewpoint from the top of the cage, we rendered seven different viewpoints per action and avatar configuration using V-Ray for Maya (v5.20.02). In line with rendered videos, we adjusted real videos to square format and dropped every third frame. Given the significant influence of the face on eye fixations [71,72], visible faces were blurred using a Fast Box Blur filter in Adobe After Effects 23.1.0 (https://www.adobe.com/de/products/aftereffects.html). Additionally, parts of the real video showing researchers or body reflections were blurred, ensuring equivalent blurred areas in the rendered videos. The mean luminance of all videos and images was equated, taking into account the gamma function of the display using custom written software.

### Eye tracking

*Setup and paradigm.* During the experiment, subjects were seated in a primate chair with their heads fixed. For eye tracking, we used an infrared eye tracker (1,000 Hz EyeLink CL, SR Research) sampled at 2,000 Hz in front of 22.5-inch, high-resolution LCD screen (VIEWPixx, 1920x1200 pixels, 120 Hz refresh rate), specifically designed for eye fixation experiments. To map the monkey’s gaze to the image’s pixel space, we conducted a calibration experiment at the beginning of each recording session. In this experiment, a red square fixation target (size = 0.2°) was presented on a gray background at 13 different locations, specified by the x, y coordinates in percentages of the display extent: [10,5], [50,5], [90,5], [35,30], [65,30], [10,50], [50,50], [90,50], [35,70], [65,70], [10,95], [50,95], [90,95].

In each trial, the target appeared at a new location, and the monkey received a juice reward for making a saccade towards the target and maintaining fixation for either 800 (for subjects: C, J, L, N, and O) or 1,000 ms (for monkey G, H, and T). The duration of fixation varied depending on the subject’s level of training. On average, we collected 11.9 valid trials at each location, with a minimum of 6 trials. The medians of these fixations at each location were then used to transform gaze coordinates into pixel coordinates, using second-order polynomial transformation (fitgeotrans, MATLAB R2020b). In the subsequent free-viewing test all stimuli had a size of 13.5 × 13.5°, or 500 × 500 pixels, and were presented in a random order for 2.4 s with an inter-stimulus interval of 400 ms. Since the stimuli covered only a part of the entire screen for presentation, a stimulus was centered on the screen and displayed on a gray background having the same luminance level as used during calibration.

There was no fixation target, and the monkeys received a juice reward as long as their gaze remained on the screen, regardless of where they were looking. During a block, each stimulus was presented once. In case the monkey’s gaze left the screen area, the stimulus remained on the screen and was not repeated during the same block to minimize the influence of familiarity on the viewing behavior. At least three runs were included from each subject in the analyses, all recorded on the same day. The start and end of the stimulus presentation were signaled by a photodiode detecting luminance changes of a small square located in the corner of the display (invisible to the animal) and placed within the same frame as the stimulus events. Stimulus presentation, event timing, and juice delivery were controlled by an in-house-developed digital signal processing-based computer system, which also sampled the photodiode signal besides vertical and horizontal eye analog signals.

*Analysis.* TrackAnything [61] (https://github.com/gaomingqi/Track-Anything) was used to define and track appropriate regions for the eye tracking analysis. This software ran locally on a machine equipped with an NVIDIA GeForce RTX 3090. We tracked masks for all real and avatar stimuli videos. Avatar masks were also used for its degraded variants, since fur-less avatars covered the same or less area in the image. For static images, we extracted the relevant masks out of the segmented videos. eye tracking data from individual subjects were then visually inspected in MATLAB R2021b to define proper values for fixation analysis with the toolbox EyeMMV [73]. The spatial constraint parameters for the clustering of the fixations were both set to $\sim 0.46^{\circ }$ in visual degrees. We required a minimum fixation duration of 180 samples or 90 ms, in line with literature on monkey eye tracking, where fixation duration thresholds varied from 50–150 ms [21,74–77].

In line with previous studies investigating the uncanny valley [21,41,55,56], we defined two measures for a quantification of differences between the conditions in the experiment: (1) the mean fixation duration; and (2) the number of fixations that fall into dynamic or static body masks, where in videos the fixation was required to be within body masks for the whole fixation duration to be accepted. For displaying fixations on stimuli (Fig 3c), we added up the Gaussian kernels ($\sigma =9\text{ pixels }\hat{=}\;0.244^{\circ }$) at the eye fixation locations, weighted by their respective fixation durations.

### Low-level visual feature control

To control for influences of low-level visual features that could explain the uncanny valley, we performed statistical analysis on the following features extracted for both images and videos of the behavioral experiment. Features were computed on a per-image basis, whereas feature values for video stimuli were averaged across frames.

*RMS Contrast.* Given a grayscale image *I* with mean pixel intensity $\bar{I}$, the root-mean-square (RMS) contrast was computed as:


$$\begin{array}{c} RMS\;Contrast=\sqrt{\frac{1}{N}\sum_{i=1}^{N}{\left(I_{i}-\bar{I}\right)}^{2}} \end{array}$$

*Edge counts*. Edges were detected and counted using the Canny edge detector [78], as implemented in OpenCV (v4.5.5.62), with lower and upper threshold values of 100 and 200, respectively.

*Spatial frequency.* Spatial frequency content was quantified using the two-dimensional Fast Fourier Transform (FFT) of grayscale images. Let *F*(*u*,*v*) denote the Fourier transform of image *I*. The power spectrum was computed as:


$$\begin{array}{c} P(u,v)=|F(u,v){|}^{2} \end{array}$$

Low- and high-spatial-frequency energy were defined as the normalized spectral power within radial frequency bands of *r* < 0.2 and *r* > 0.5, respectively, where *r* denotes normalized distance from the center of the frequency spectrum.

*Saliency entropy and area.* Visual saliency maps were computed using the Itti and Koch saliency model [79], as implemented in the SaliencyToolbox [80] using Matlab. Saliency maps were then normalized prior to analysis. The saliency entropy was computed as the Shannon entropy of the normalized saliency distribution:


$$\begin{array}{c} H(S)=-\sum_{i=1}^{N}p_{i}\log_{2}(p_{i}) \end{array}$$

where


$$\begin{array}{c} p_{i}=\frac{S_{i}}{\sum_{j=1}^{N}S_{j}} \end{array}$$

denotes the normalized saliency value at pixel *i* and *N* is the total number of pixels. Higher entropy values indicate a more spatially diffuse saliency distribution, whereas lower values indicate more spatially concentrated saliency.

Salient Area was quantified as the proportion of pixels whose normalized saliency exceeded pre-defined thresholds (0.50, 0.65, and 0.80).

### Statistical analysis

*Plots.* Point plots show arithmetic mean values as dots, with vertical lines depicting the standard error for image coverage (except for individuals), and .95 confidence intervals otherwise. Box plots illustrate the median along with interquartile range (IQR), where whiskers extend to 1.5× the IQR. The arithmetic means are shown as white circles. Statistical significant pair-wise differences are indicated by horizontal lines above relevant groups, accompanied by asterisks: $p^{*}<0.05$, $p^{**}<0.01$, and $p^{***}<0.001$.

*Tracking.* Euclidean error plots show mean marker Euclidean distances. When referring to a specific training configuration in the text, which is characterized by the algorithm, cameras, and labeled keyframes used for training, we present the Euclidean error in terms of their mean and standard deviation.

*Behavioral experiment.* To model fixation counts within the whole image and the monkey sihouette, we additionally constructed GLMs with Poisson and negative binomial distributions using the package MASS 7.3.60 in R. In line with [56], we also constructed generalized linear mixed effect models (GLMMs) with Poisson and negative binomial distributions with the package lme4 1.1.33. Subject differences in fixation counts were accounted for by a random intercept for each individual. We employed negative binomial GLMs as these showed no effects of under- and over-dispersion, unlike all other models. Therefore, we tested fitted model residuals and simulated residuals with the function testDispersion() and inspected Q-Q plots to assess model fit, using the package DHARMa 0.4.6. Afterwards, we computed LRTs with the package lmtest 0.9.40 for different nested parameter combinations to identify interactions within predictor variables.

For identifying trends in fixation counts across trials for single individuals, we fitted separate linear models using the lm() function from the stats package. For each individual, fixations were modeled as a function of trial number. Slope estimates and associated *p*-values were extracted to assess the direction and significance of trial-wise trends.

For nonparametric testing, we used the Friedman test from the scipy.stats 1.9.0 package in Python 3.9.7. These tests were based on within-subject centered data. Testing the valley’s minima, maxima and U-shape was done with the two-lines test app 0.52 [62] in R.

## Supporting information

S1 Video Comparison of the real and animated turning action. The movie presents a sample camera view of the submissive turning movement as shown to the macaque observers, with faces blurred. The scene and camera movements replicate real-world measurements. The real footage appears on the left, while the animated version is displayed on the right.
(MP4)

S2 Video Comparison of the real and animated walking action. The movie presents a sample camera view of the walking sequence as shown to the macaque observers, with faces blurred. The scene and camera movements replicate real-world measurements. The real footage appears on the left, while the animated version is displayed on the right.
(MP4)

S3 Video Methods for action interpolation in comparison. The 3D turning movement interpolation based on six labeled frames for training different algorithmic approaches using six cameras. It shows simultaneously the results of structurally refined and optimized triangulation with DLC + Anipose, as well as the volumetric method, DANNCE.
(MP4)

S1 Appendix Supplementary figures and tables. Contains Fig A–C and Tables A–G, including extended results for single-action interpolation in macaques and humans, extended results of the uncanny valley experiment, low-level visual feature controls, regression model results, trend tests, likelihood-ratio tests, and nonlinearity/U-shape analyses.
(PDF)

## Abbreviations

AOIs: areas of interest
COM: center of mass
DANNCE: Dimensional Aligned Neural Network for Computational Ethology
DLC: DeepLabCut
DOF: degrees of freedom
FFT: Fast Fourier Transform
FOV: field of view
FPS: frames per second
GLMMs: generalized linear mixed effect models
GLMs: generalized linear models
IPJTD: instantaneous per-joint temporal deviation
IQR: interquartile range
LPS: labeled frames per second
LRTs: likelihood-ratio tests
MPJPE: mean per-joint position error
MPJTD: mean per-joint temporal deviation
NHP: nonhuman primate
RANSAC: Random Sample Consensus
RMS: root-mean-square.
