# Supplementary material for: Realistic monkey body animation reveals an uncanny valley in macaque body perception
Source: PLoS Biol. 2026 Jul 14;24(7):e3003880. doi: 10.1371/journal.pbio.3003880 (PMC13367704; doi:10.1371/journal.pbio.3003880)
Supplement: S1 Appendix — Contains Fig A–C and Tables A–G, including extended results for single-action interpolation in macaques and humans, extended results of the uncanny valley experiment, low-level visual feature controls, regression model results, trend tests, likelihood-ratio tests, and nonlinearity/U-shape analyses. (PDF) [file pbio.3003880.s004.pdf]

S1 Appendix

Realistic monkey body animation reveals an uncanny valley in macaque body perception

Lucas M. Martini*, Anna Bognár, Rufin Vogels, and Martin A. Giese*

Corresponding author(s): martin.giese@uni-tuebingen.de; lucas.martini@uni-tuebingen.de

Supplementary Figures

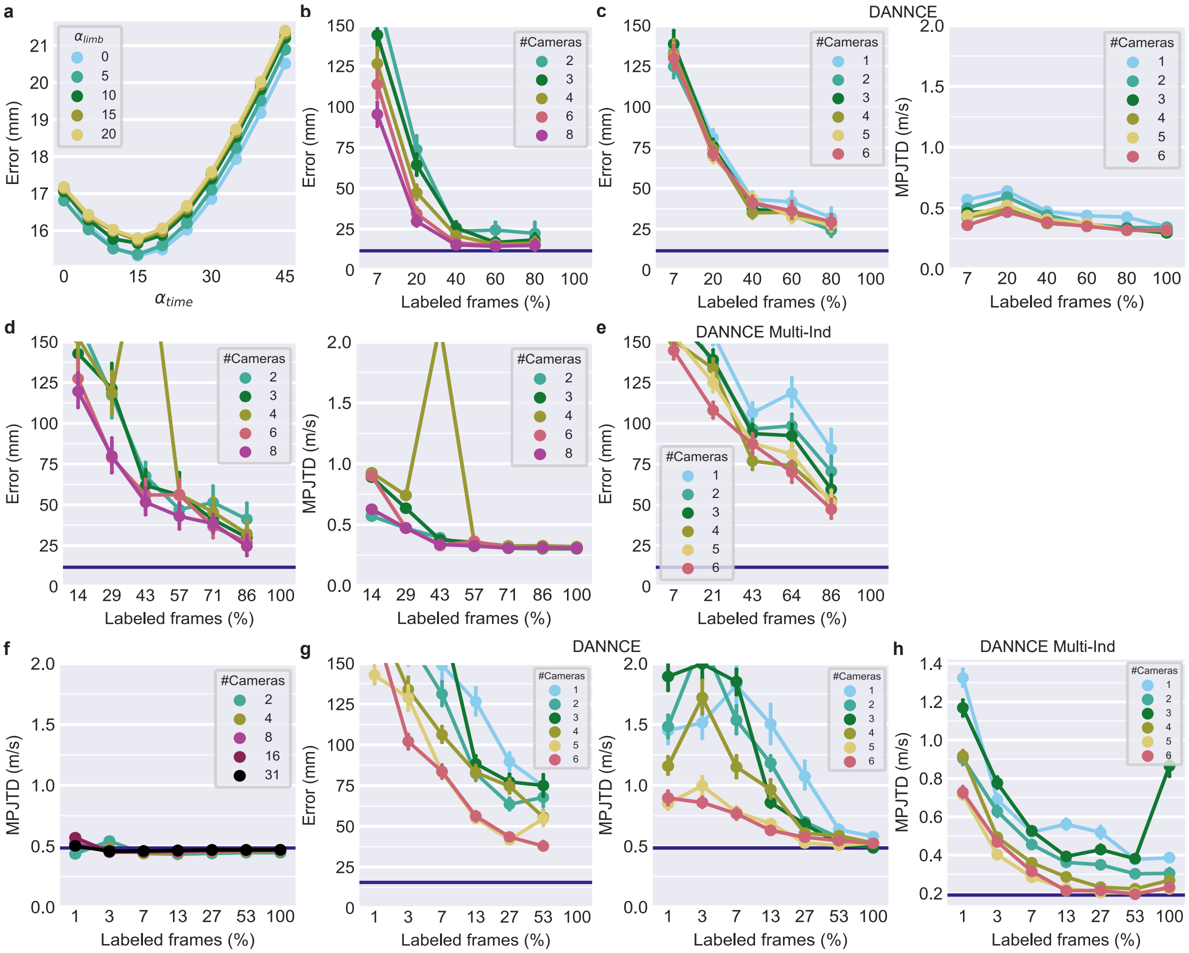

Fig A. Related to Fig 2: Extended results for single action interpolation in macaques and humans. a, Optimization of Anipose's spatiotemporal parameters for 40% or six frames and eight cameras for the densely labeled submissive action. **b,** Euclidean error on test frames of the submissive action that were not part in the training of labeled frames for spatiotemporally refined triangulation with Anipose. Results are shown for different numbers of cameras, and varying the amount of labeled keyframes used for training. The violet horizontal line indicates the spatial resolution (for macaques in **b-e**: 11.72mm) of the comparative volumetric approach, DANNCE. **c,** Euclidean error and MPJTD for the submissive action with DANNCE using up to a maximum of six cameras. DANNCE results are limited to six camera views because the pre-trained weights used for transfer learning were trained with six camera inputs. **d,** Euclidean error (left) and MPJTD (right) for the walking action with DLC and spatiotemporally refined triangulation. **e,** Euclidean error for the multi-animal action with DANNCE. **f,** MPJTD for DLC with spatiotemporally optimized triangulation for the single-actor human action. Violet horizontal lines in MPJTD plots in panels **f-h** show the ground-truth mean marker speeds for the single (0.49m/s) and group (0.19m/s) actions. The spatial resolution for human action interpolation with DANNCE is 15.625mm. **g,** Euclidean error (left) and MPJTD (right) for the entire sequence of the single-actor human action using DANNCE. **h,** MPJTD for the multi-actor action prediction with DANNCE. The deviations from the overall trends in panels **d** and **h** are attributable to conditions with small amounts of test data and random outliers that were not reproduced in repeated simulations. The data underlying this Figure can be found in `https://doi.org/10.5281/zenodo.20490287`.

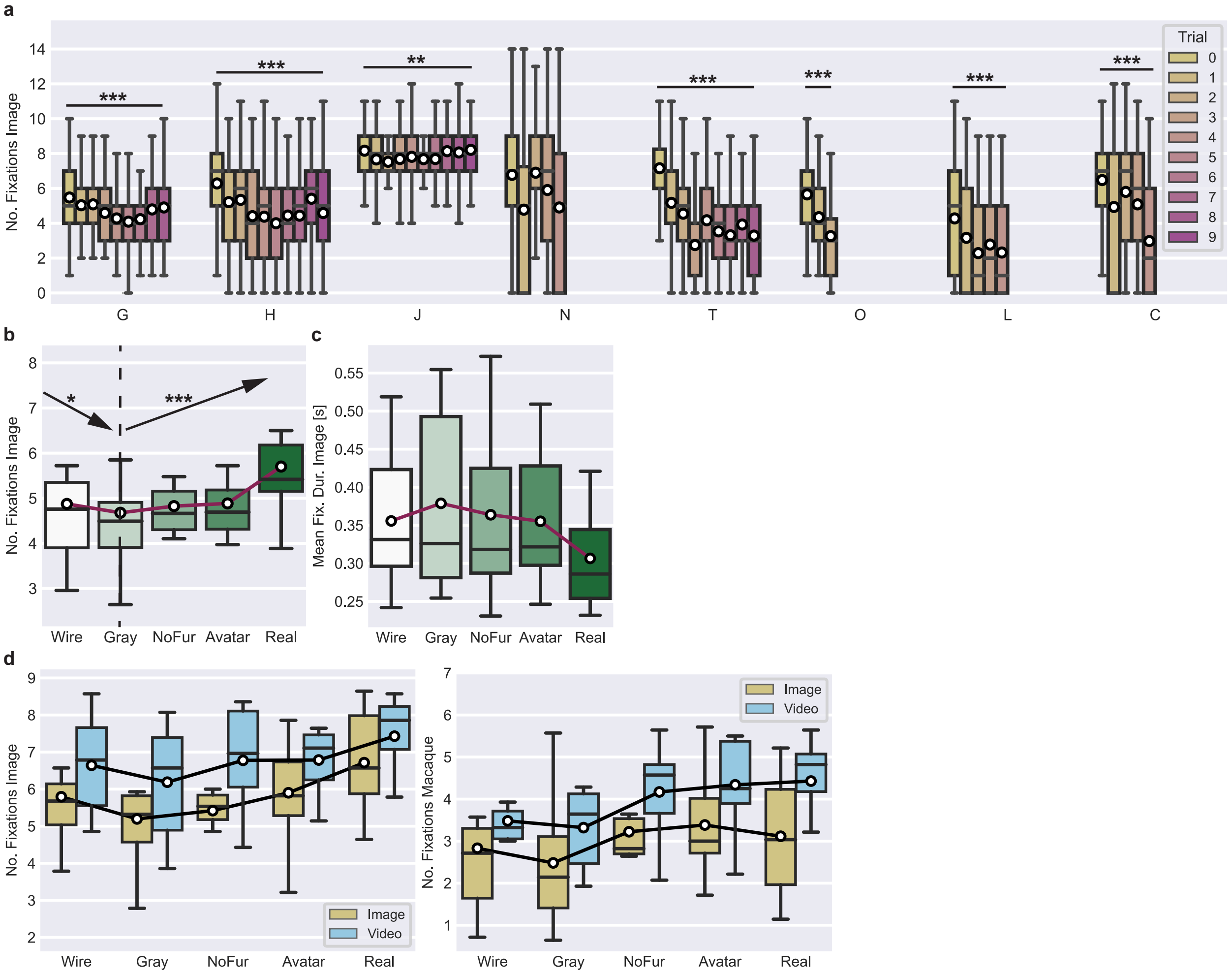

Fig B. Related to Fig 4: Extended results of the uncanny valley experiment. a, Number of image fixations over repeated trials for the eight monkeys. Significant influences of the trial number in modeling fixations counts are shown above. Box plots show the median with interquartile range (IQR), whiskers at 1.5× the IQR, and arithmetic means are shown as white circles. In **b,c**, these arithmetic means are connected by a red line. Statistically significant slopes are indicated by asterisks: $p^{*} < 0.05$, $p^{**} < 0.01$ and $p^{***} < 0.001$. **b,** The number of image fixations as a function of realism or Render Type including all trials. The Two-lines test [1] was performed on centered data without aggregation, and we indicate the slopes direction by arrows. The vertex of the valley is shown as a dashed vertical line. **c,** Box plot of the mean fixation duration on the image as a function of Render Type, for the first trials only. **d,** The number of fixations on the image (left), and within the macaque silhouette (right) as function of Render Type, but split by Presentation Type (still images vs. videos). The plots show first trials, and arithmetic means per condition (white circles) are connected by black lines. The data underlying this Figure can be found in `https://doi.org/10.5281/zenodo.20490287`.

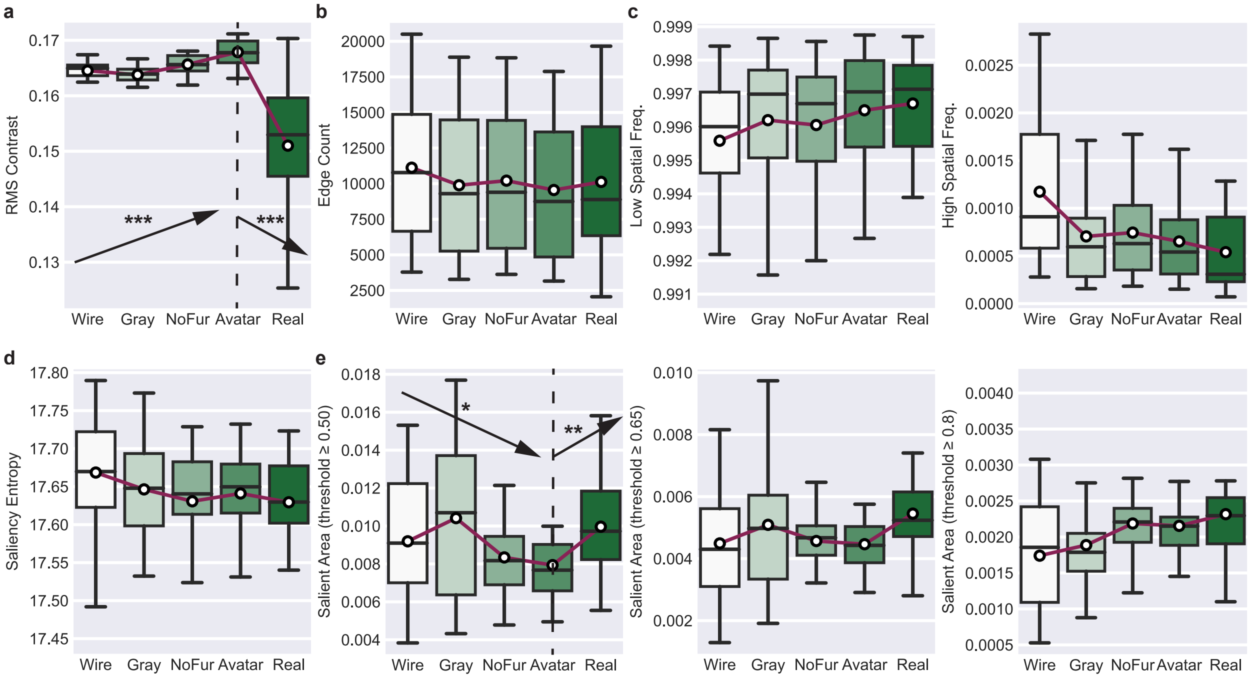

Fig C. Related to Fig 4: Low-level visual feature control in the uncanny valley experiment. Different low-level visual features do not explain or follow the monkeys' behavioral response. **a,** Root-mean-square (RMS) contrast of grayscale images. Significant influences of the trial number in modeling fixations counts are shown above. Box plots show the median with interquartile range (IQR), whiskers at 1.5× the IQR, and arithmetic means are shown as white circles. In **a-e**, these arithmetic means are connected by a red line. Statistically significant slopes are indicated by asterisks: $p^{*} < 0.05$, $p^{**} < 0.01$ and $p^{***} < 0.001$. The Two-lines test [1] was performed, and if a U-shape was detected, we indicate the corresponding slopes' direction by arrows and show their significance. The vertex of the valley is shown as a dashed vertical line. **b,** The number of edges detected using the Canny edge detector [2]. **c,** The normalized power spectrum of a two-dimensional Fast Fourier Transform (FFT) split into low and high spatial frequencies. **d,** Entropy of saliency maps computed using the Itti & Koch saliency model [3]. **e,** Salient areas derived from the saliency maps at different thresholds (0.5, 0.65, and 0.8). The data underlying this Figure can be found in `https://doi.org/10.5281/zenodo.20490287`.

Supplementary Tables

Table A. Negative Binomial Regression model for monkey silhouette fixation counts on all trials and experimental factors. In order to analyze the image and monkey silhouette fixation counts as functions of Trial, Action, View, Presentation Type (video vs. static), and Render Type (realism), we performed a binomial regression analysis. The corresponding model summary is shown for monkey silhouette fixations in the following table:

	Coefficient	95%-confidence interval	Standarderror	*p*-value
Intercept	1.16	from 2.91 to 3.50	0.048	< 0.0001
ActionWalk	0.22	from 1.20 to 1.30	0.02	< 0.0001
ViewC	-0.023	from 0.91 to 1.05	0.036	0.53
ViewD	-0.13	from 0.82 to 0.94	0.036	0.0004
ViewE	-0.30	from 0.69 to 0.80	0.037	< 0.0001
ViewF	-0.25	from 0.73 to 0.84	0.037	< 0.0001
ViewG	-0.18	from 0.78 to 0.90	0.036	< 0.0001
ViewH	-0.31	from 0.68 to 0.79	0.037	< 0.0001
PresentationStatic	-0.15	from 0.82 to 0.89	0.02	< 0.0001
IndividualGin	-0.024	from 0.90 to 1.06	0.043	0.57
IndividualHyde	0.11	from 1.03 to 1.21	0.042	0.011
IndividualJekyll	1.05	from 2.65 to 3.10	0.039	< 0.0001
IndividualLibre	-0.73	from 0.43 to 0.53	0.053	< 0.0001
IndividualNacho	0.05	from 0.96 to 1.15	0.046	0.28
IndividualOdin	-0.11	from 0.80 to 0.99	0.054	0.039
IndividualTonic	-0.51	from 0.55 to 0.66	0.045	< 0.0001
RenderAvatar	0.0014	from 0.94 to 1.06	0.031	0.96
RenderGray	-0.18	from 0.79 to 0.89	0.032	< 0.0001
RenderReal	-0.02	from 0.92 to 1.04	0.031	0.51
RenderWireframe	-0.19	from 0.77 to 0.88	0.032	< 0.0001
Trial	-0.07	from 0.93 to 0.94	0.0041	< 0.0001

Table B. Related to panel a of Fig B: Trend tests of the number of image fixations across trials for each individual. Linear models had significant negative slopes in 6 out of 8 cases, indicating a decay in the subject's attention over trials. Significance was assessed using a Bonferroni-adjusted alpha level of 0.00625 (0.05/8 individuals).

Individual	Estimate	Std. Error	*t* value	*p* value	Sig. ($\alpha_{Bonf} = 0.00625$)
C	-0.68	0.09	-7.69	4.91e-14	Yes
G	-0.09	0.02	-4.51	7.12e-06	Yes
H	-0.11	0.02	-5.03	5.66e-07	Yes
J	0.04	0.01	3.07	2.17e-03	Yes
L	-0.43	0.08	-5.62	2.80e-08	Yes
N	-0.26	0.10	-2.59	9.69e-03	No
O	-1.20	0.12	-10.02	2.60e-21	Yes
T	-0.35	0.03	-13.51	5.83e-39	Yes

Table C. Interaction between Render Type and Presentation Type for real videos and avatars only. Likelihood-ratio tests (LRTs) for fixation counts on *monkey silhouettes* for the first trial. The influences of Render and Presentation Type (Pres.) were significant, unlike their interaction.

	Model	Criterion variable	Predictor variables	df-model	df	χ^2-statistic	p-value
LRT 1	Θ_0	MonkeyFixations	Action + View + Individual + Render	17			
	Θ	MonkeyFixations	Action + View + Individual + Render + Pres.	18			
					1	33.0	< 0.0001
LRT 2	Θ_0	MonkeyFixations	Action + View + Individual + Pres.	17			
	Θ	MonkeyFixations	Action + View + Individual + Pres. + Render	18			
					1	0.27	0.6005
LRT 3	Θ_0	MonkeyFixations	Action + View + Individual + Pres. + Render	18			
	Θ	MonkeyFixations	Action + View + Individual + Pres. + Render + Render * Pres.	19			
					1	0.9651	0.3259

Table D. Interaction between Render Type and Presentation Type. Likelihood-ratio tests (LRTs) for the *image* fixation counts for the first trial. The Render Type and Presentation Type (Pres.) were each significant for modeling, unlike their interaction.

	Model	Criterion variable	Predictor variables	df-model	df	χ^2-statistic	p-value
LRT 1	Θ_0	ImageFixations	Action + View + Individual + Render	20			
	Θ	ImageFixations	Action + View + Individual + Render + Pres.	21			
					1	41.0	< 0.0001
LRT 2	Θ_0	ImageFixations	Action + View + Individual + Pres.	17			
	Θ	ImageFixations	Action + View + Individual + Pres. + Render	21			
					4	35.66	< 0.0001
LRT 3	Θ_0	ImageFixations	Action + View + Individual + Pres. + Render	21			
	Θ	ImageFixations	Action + View + Individual + Pres. + Render + Render * Pres.	25			
					4	3.05	0.5502

Table E. Results of testing the non-linearity or U-shapeness of image fixations and durations using the Two-lines test [1] for different number of trials. A U-shape is detected if and only if around the vertex two significant slopes with opposite directions are present (conjunction testing, $\alpha_{Constituent} = \alpha_{Joint} = 0.05$ in [1], cf. [4]).

Measure	Trials	U-shape detected	Vertex	Slope 1 (z, p)	Slope 2 (z, p)
Image Fixations	First	Yes	Gray	(-2.42, 0.0156)	(6.1, < 0.0001)
Mean ImageFixation Dur.	First	No	Color	(0.43, 0.6689)	(-2.96, 0.0031)
ImageFixations	All	Yes	Gray	(-2.44, 0.0147)	(9.34, < 0.0001)
Mean ImageFixation Dur.	All	Yes	Gray	(1.97, 0.0487)	(-8.37, < 0.0001)

Table F. Interaction between Render Type and Presentation Type. Likelihood-ratio tests (LRTs) for fixation counts on *monkey silhouettes* for the first trial. The influences of Render and Presentation Type (Pres.) were significant, unlike their interaction.

	Model	Criterion variable	Predictor variables	df-model	df	χ^2-statistic	p-value
LRT 1	Θ_0	MonkeyFixations	Action + View + Individual + Render	20			
	Θ	MonkeyFixations	Action + View + Individual + Render + Pres.	21			
					1	58.44	< 0.0001
LRT 2	Θ_0	MonkeyFixations	Action + View + Individual + Pres.	17			
	Θ	MonkeyFixations	Action + View + Individual + Pres. + Render	21			
					4	35.40	< 0.0001
LRT 3	Θ_0	MonkeyFixations	Action + View + Individual + Pres. + Render	21			
	Θ	MonkeyFixations	Action + View + Individual + Pres. + Render + Render * Pres.	25			
					4	1.60	0.8086

Table G. Results of testing the non-linearity or U-shapeness of different low-level visual feature descriptors using the Two-lines test [1] . A U-shape is detected if and only if around the vertex two significant slopes with opposite directions are present (conjunction testing, $\alpha_{Constituent} = \alpha_{Joint} = 0.05$ in [1], cf. [4]). Different saliency area thresholds are indicated by @ followed by the normalized saliency threshold value. Only two of the eight analyzed low-level feature measures showed a significant U-shape, and neither was centered on the Gray Avatar condition that elicited the behavioral uncanny valley.

Measure	U-shape detected	Vertex	Slope 1 (z, p)	Slope 2 (z, p)
RMS Contrast	Yes	Avatar	(6.5, $<$ 0.0001)	(-6.15, $<$ 0.0001)
Edge Counts	No	Gray	(-0.9, 0.3666)	(0.01, 0.9921)
Low Spatial Frequency	No	Avatar	(1.55, 0.1212)	(0.45, 0.6500)
High Spatial Frequency	No	Color	(-2.47, 0.0136)	(-1.69, 0.0916)
Saliency Entropy	No	Avatar	(-1.71, 0.0863)	(-0.73, 0.4638)
Saliency Area @0.50	Yes	Avatar	(-2.44, 0.0148)	(3.16, 0.0016)
Saliency Area @0.65	No	Avatar	(-0.46, 0.6445)	(2.92, 0.0035)
Saliency Area @0.80	No	Avatar	(2.86, 0.0042)	(1.11, 0.2675)

References

[1] Simonsohn U. Two Lines: A Valid Alternative to the Invalid Testing of U-Shaped Relationships With Quadratic Regressions. Advances in Methods and Practices in Psychological Science. 2018;1(4):538-55.

[2] Canny J. A Computational Approach to Edge Detection. IEEE Transactions on Pattern Analysis and Machine Intelligence. 1986;PAMI-8(6):679-98.

[3] Itti L, Koch C. A saliency-based search mechanism for overt and covert shifts of visual attention. Vision Research. 2000;40(10):1489-506.

[4] Berger RL. Multiparameter Hypothesis Testing and Acceptance Sampling. Technometrics. 1982;24(4):295-300.
